# Assessing the Inflow Segment of a Hemodialysis Access: The Fogarty Balloon Occlusion Technique

**DOI:** 10.7759/cureus.41534

**Published:** 2023-07-07

**Authors:** Mona J Malik, Rema J Nabi

**Affiliations:** 1 Internal Medicine, University of California, Riverside, Riverside, USA; 2 Vascular Surgery, University of Houston, Houston, USA

**Keywords:** vascular surgery, fistulogram, arteriovenous access, graft, fistula, dialysis access

## Abstract

Dialysis access maintenance often requires a fistulogram or shuntogram of arteriovenous access. Assessment of the arterial inflow segment and arterial anastomosis is often a critical portion of the procedure. Retrograde occlusive angiography (ROA) is often used to properly assess the inflow. Manual compression using finger compression or a hemostat is often described in the literature. The Fogarty balloon occlusion technique using a 4-Fr Fogarty catheter balloon (Henry Shein) is a simple and cost-effective method that preserves image quality and decreases radiation exposure in retrograde occlusive angiography.

## Introduction

The preservation of functional vascular access in patients on hemodialysis can be quite challenging for the healthcare team. Performing a fistulogram is a crucial step before an intervention is carried out to salvage hemodialysis access. In addition to assessing the outflow segment of the fistula or graft, it is important to visualize arterial anastomosis and inflow segments during the fistulogram. Retrograde occlusive angiography (ROA) is commonly used to depict the inflow segment of the arteriovenous fistula (AVF) or graft (AVG) [1,2]. ROA can be performed by manually compressing the outflow segment or occluding the outflow segment with a 4-Fr Fogarty catheter balloon (Henry Shein) while injecting the contrast. The manual occlusion technique is more commonly used and discussed in the literature [3]. The purpose of this article is to describe the balloon occlusion technique and highlight its advantages.

## Technical report

**Figure 1:**
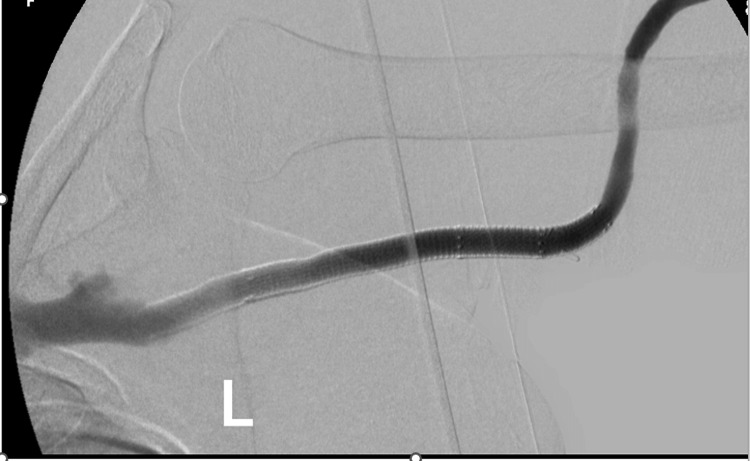
Fistulogram with the assessment of venous outflow

The patient is placed in the supine position with the arm of interest extended on a surgical table. The patient is sedated by the anesthetist, and local anesthesia is administered. Access to the fistula or graft is obtained, and the outflow component of the dialysis circuit is investigated with angiography in a routine fashion (Figure 1).

**Figure 2:**
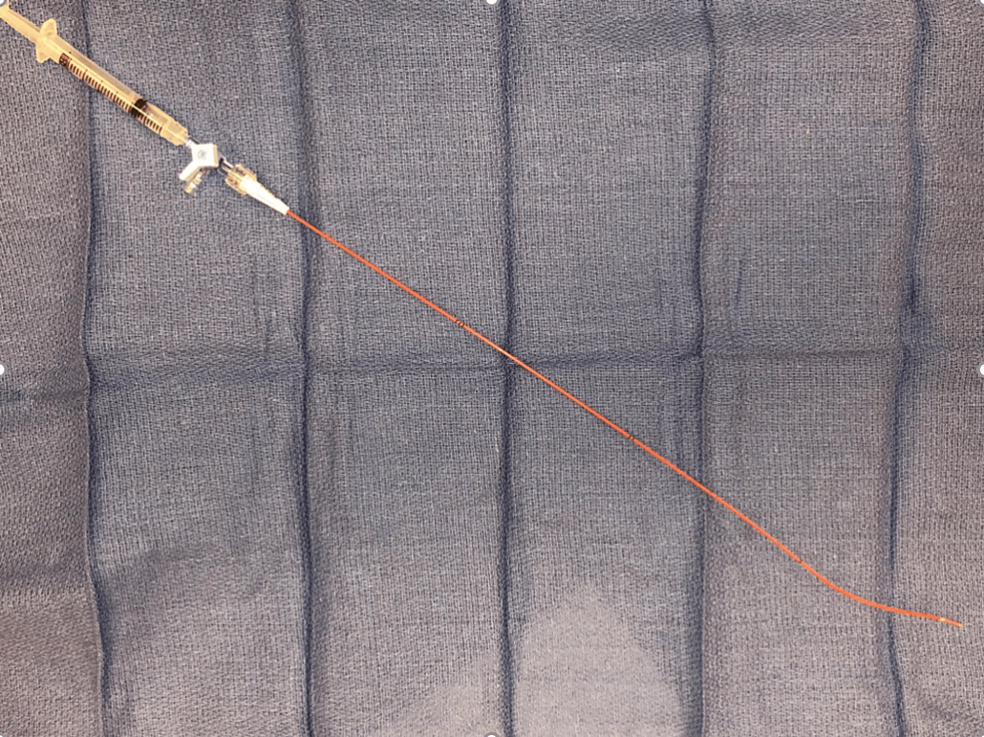
4-Fr Fogarty balloon catheter attached to a three-way stop cock

After obtaining images of the complete venous outflow, a contrast-filled (with 50% saline/contrast) 4-Fr Fogarty balloon catheter attached to a three-way stop cock is inserted into the fistula (Figure 2).

**Figure 3:**
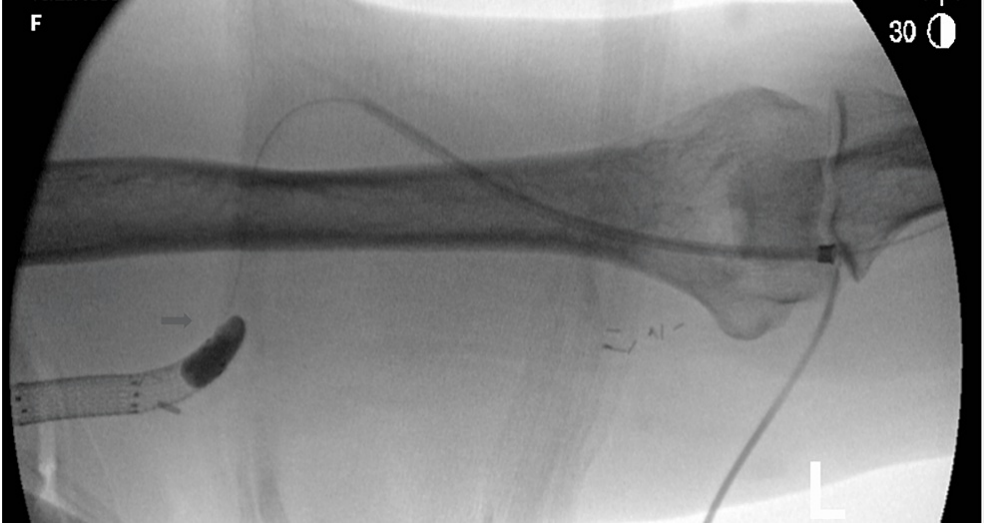
Fogarty balloon inflated to occlude the venous outflow tract

The balloon is inflated to occlude the venous outflow tract (Figure 3).

**Figure 4:**
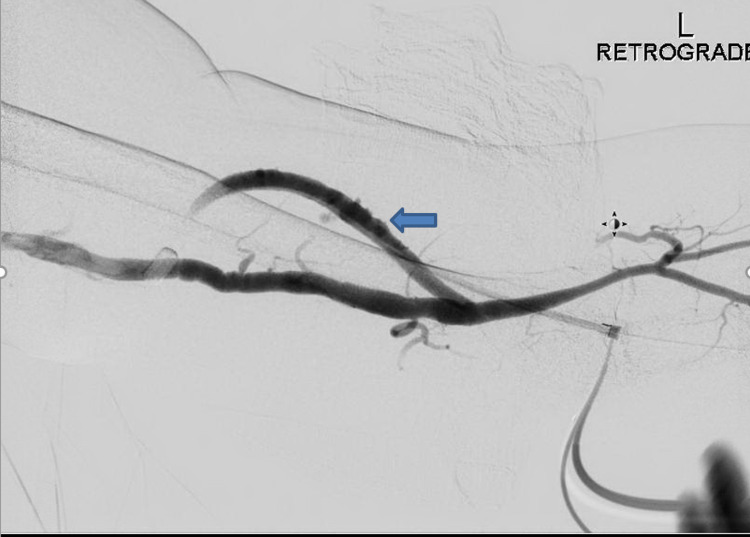
Arterial inflow segment evaluation (arrow)

The contrast is injected, and there is a reflux of contrast into the inflow segment. The anastomotic and inflow/arterial segments of the AVG/AVF are assessed (Figure 4).

During the manual occlusion technique, manual pressure is applied to the fistula or graft, and when released, it will provide arterial inflow imaging that serves as a road map for interventions.

**Figure 5:**
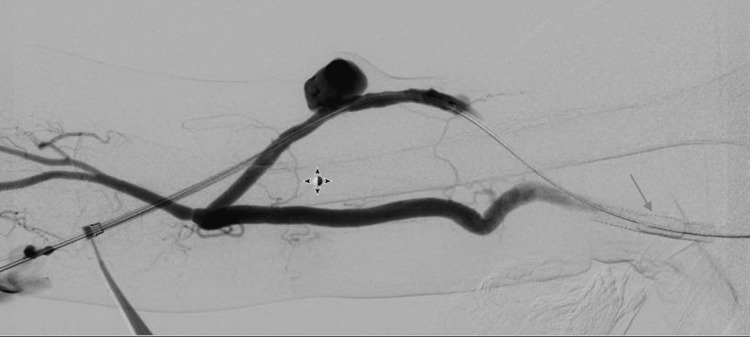
Retrograde angiography with balloon occlusion of the venous outflow segment with delineation of aneurysm in the arterial inflow segment

**Figure 6:**
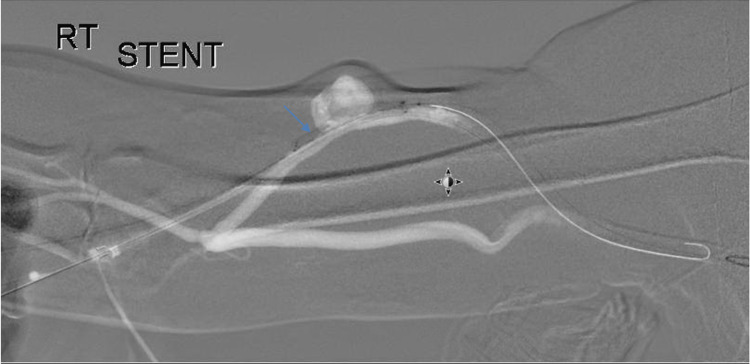
Road map being used to treat the aneurysmal segment with stent graft

Balloon occlusion may serve as a very valuable asset in maintaining a road map during coil embolization of the branches and aneurysm repair in dialysis access procedures (Figures 5, 6).

A decision is then made on the specific type of interventional procedure to be performed. Once the intervention has been performed, a completion fistulogram is carried out. After the fistulogram, the sheath and the catheter are removed. Hemostasis is achieved in a standard fashion.

## Discussion

A diagnostic fistulogram should be completed from the arterial inflow segment to the right atrium to fully evaluate the AVG or AVF before performing an intervention. Problems associated with the arterial/inflow and anastomotic segment are responsible for a significant proportion of AVFs and AVGs dysfunction [4,5]. Stenosis along the arterial limb may coexist with stenosis involving the venous limb and may be overlooked if the arterial inflow is not assessed. The techniques used in delineating the inflow segment of an AVG/AVF include direct arteriography (DA) and retrograde occlusive angiography (ROA). In DA, the inflow segment is depicted by injection of contrast following a direct arterial puncture. DA is associated with a high risk of complications [6]. Complications associated with DA include arterial thrombosis, hand ischemia, hematoma, nerve injuries, and spasm [1,7]. In ROA, the venous limb/graft is cannulated, and the inflow segment is depicted by reflux of injected contrast following temporary occlusion of the outflow tract. ROA has been associated with complications such as vessel spasms and vessel dilatation. Chan et al. [8] reported that ROA may fail to identify some inflow lesions. In ROA, the outflow segment can be occluded by extrinsic (manual) compression or by balloon occlusion. Extrinsic compression is the technique traditionally used by most vascular surgeons, and most of the literature on ROA discusses solely the extrinsic compression technique. The balloon occlusion technique has some advantages over extrinsic compression; it is, therefore, imperative to discuss these advantages.

With the manual compression technique, the operator may be exposed to more radiation as he uses his hand to compress the outflow segment during the fistulogram [2]. This risk is reduced in the balloon occlusion technique since manual compression is not needed. The issue of radiation exposure and attenuation is of significant concern among vascular surgeons. It is recommended that every approach to reduce radiation exposure be implemented meticulously [9]. Heye et al. [10] noted that the position of the operator’s hand relative to the X-ray tube was the major determinant of the dose of radiation exposure.

The other option for attenuating radiation exposure to the operator would be the use of lead gloves. It is, however, interesting to note that the unit price of a Fogarty balloon catheter is about $30, while the price for a pair of lead gloves used in manual compression costs about $80, thus making the balloon occlusion technique a relatively cheaper alternative barring other costs. While the unit price difference may not be overly significant, the importance of cost-effective treatment models cannot be overemphasized in this era. Vascular access maintenance including diagnostic and therapeutic procedures constitutes a significant portion of the healthcare costs of patients on hemodialysis [11-13].

## Conclusions

Extrinsic compression of the venous outflow limb is the traditional approach for retrograde occlusive angiography. This method has been used to assess the arterial inflow limb of the arteriovenous fistula or graft in dialysis maintenance procedures. We present a simple new technique utilizing Fogarty balloon occlusion that minimizes radiation exposure and is a safe, cost-effective, and more efficient alternative for the assessment of arterial inflow in dialysis maintenance procedures.

## References

[1] Le L, Brooks A, Donovan M, Smith TA, Sternbergh WC 3rd, Bazan HA (2015). Transradial approach for percutaneous intervention of malfunctioning arteriovenous accesses. J Vasc Surg.

[2] Kandarpa K, Machan L (2011). Handbook of interventional radiologic procedures, fourth edition.

[3] Salman L, Asif A, Beathard GA (2009). Retrograde angiography and the risk of arteriovenous fistula perforation. Semin Dial.

[4] Beathard GA, Arnold P, Jackson J, Litchfield T (2003). Aggressive treatment of early fistula failure. Kidney Int.

[5] Asif A, Gadalean FN, Merrill D, Cherla G, Cipleu CD, Epstein DL, Roth D (2005). Inflow stenosis in arteriovenous fistulas and grafts: a multicenter, prospective study. Kidney Int.

[6] Duijm LE, Overbosch EH, Liem YS (2009). Retrograde catheterization of haemodialysis fistulae and grafts: angiographic depiction of the entire vascular access tree and stenosis treatment. Nephrol Dial Transplant.

[7] Murphy EA, Ross RA, Jones RG (2017). Imaging in vascular access. Cardiovasc Eng Technol.

[8] Chan MR, Chhokar VS, Young HN, Becker BN, Yevzlin AS (2011). Retrograde occlusive arteriography of hemodialysis access: failure to detect inflow lesions?. Semin Dial.

[9] Attigah N, Oikonomou K, Hinz U, Knoch T, Demirel S, Verhoeven E, Böckler D (2016). Radiation exposure to eye lens and operator hands during endovascular procedures in hybrid operating rooms. J Vasc Surg.

[10] Heye S, Maleux G, Oyen RH, Claes K, Kuypers DR (2012). Occupational radiation dose: percutaneous interventional procedures on hemodialysis arteriovenous fistulas and grafts. Radiology.

[11] Coentrão LA, Araújo CS, Ribeiro CA, Dias CC, Pestana MJ (2013). Cost analysis of hemodialysis and peritoneal dialysis access in incident dialysis patients. Perit Dial Int.

[12] Allon M, Dinwiddie L, Lacson E Jr, Latos DL, Lok CE, Steinman T, Weiner DE (2011). Medicare reimbursement policies and hemodialysis vascular access outcomes: a need for change. J Am Soc Nephrol.

[13] Manns B, Tonelli M, Yilmaz S (2005). Establishment and maintenance of vascular access in incident hemodialysis patients: a prospective cost analysis. J Am Soc Nephrol.

